# Evidence for alterations in fixational eye movements in glaucoma

**DOI:** 10.1186/s12886-018-0870-7

**Published:** 2018-08-03

**Authors:** Giovanni Montesano, David P. Crabb, Pete R. Jones, Paolo Fogagnolo, Maurizio Digiuni, Luca M. Rossetti

**Affiliations:** 1 ASST Santi Paolo e Carlo, University of Milan, 20142 Milan, Italy; 2 City, University of London, Optometry and Visual Sciences, Northampton Square, EC1V 0HB, London, UK

**Keywords:** Fundus perimetry, Fixation, Glaucoma, Eye movements, Visual field

## Abstract

**Background:**

Fixation changes in glaucoma are generally overlooked, as they are not strikingly evident as in macular diseases. Fundus perimetry might give additional insights into this aspect, along with traditional perimetric measures. In this work we propose a novel method to quantify glaucomatous changes in fixation features as detected by fundus perimetry and relate them to the extent of glaucomatous damage.

**Methods:**

We retrospectively analysed fixation data from 320 people (200 normal subjects and 120 with glaucoma) from the Preferred Retinal Locus (PRL) detection of a Compass perimeter. Fixation stability was measured as Bivariate Contour Ellipse Area (BCEA), and using two novel metrics: (1) Mean Euclidean Distance (MED) from the Preferred Retinal Locus, and (2) Sequential Euclidean Distance (SED) of sequential fixation locations. These measures were designed to capture the spread of fixation points, and the frequency of position changes during fixation, respectively.

**Results:**

In the age corrected analysis, SED was significantly greater in glaucomatous subjects than controls (*P =* 0.002), but there was no difference in BCEA (*P =* 0.15) or MED (*P =* 0.054). Similarly, SED showed a significant association with Mean Deviation (*P* <  0.001), but neither BCEA nor MED were significantly correlated (*P* > 0.14 for both).

**Conclusion:**

Changes in the scanning pattern detected by SED are better than traditional measures of fixation spread (BCEA) for describing the changes in fixation stability observed in glaucoma.

## Background

Primary Open Angle Glaucoma (POAG) is a progressive optic neuropathy usually associated with increased intraocular pressure (IOP), progressive damage to the visual field and characteristic changes in the optic nerve and the inner retina [1]. Both structural and functional measurements are employed in the diagnosis of POAG, most notably Optical Coherence Tomography (OCT) for the structural assessment, and Visual Field testing (VF) for the functional assessment [2].

VF testing (static white-on-white perimetry), measures the differential light sensitivity (DLS) by presenting light points of variable intensity, in order to assess patient’s detection threshold at various retinal locations. VF testing is one of the most useful means of diagnosing and staging glaucoma and assessing progression [3]. However, classical VF testing requires the patient to be able to fixate a central target throughout the test. To assess the accuracy of the exam, the Humphrey Field Analyzer (HFA) is able to report fixation performances on the final printout both as a descriptive plot from a simple eye tracker and as an estimate of fixation loss with the classical Heijl-Krakau technique [4].

Fundus Automated Perimetry (FAP), also known as microperimetry, has been introduced to allow reliable testing in patients with central vision impairment, such as in macular degenerations, who are usually not able to maintain a stable central fixation. FAP uses continuous infrared Scanning Laser Ophthalmoscope (SLO) imaging to track the retina and compensates for eye movements during the presentation of the stimuli [5]. Despite this, very unsteady fixation is still likely to be a confounding factor [6], due to limited temporal resolution or poor image quality during tracking.

Furthermore, even with a perfect control of fixation shifts in perimetry, fixation data might contain useful information that can be exploited [7]. Recent works have analysed fixational movements during central static fixation with microperimetry [8, 9], reporting partially contrasting results. At the same time there has been an interest in eye movement behaviour in people with glaucoma and how it differs from visually healthy peers when undergoing different visual tasks [10–13]. A better understanding of fixation alterations in glaucomatous patients might be useful to improve visual field testing accuracy by gathering additional functional information on individual VF loss [14].

Some attempts have been made to produce a quantitative evaluation of the gaze tracking data [15]. The Bivariate Contour Ellipse Area (BCEA) [16] is commonly used to quantify the spatial extent of fixations collected over a period of time. The BCEA has limitations since it assumes that fixations are normally distributed in space. Moreover, the BCEA disregards the temporal sequence of fixation movements [17].

In short, fixation analysis has the potential to be an additional tool in characterizing functional changes in glaucoma, but current tools might be not adequate to characterize fixation features to their full extent.

**Fig. 1:**
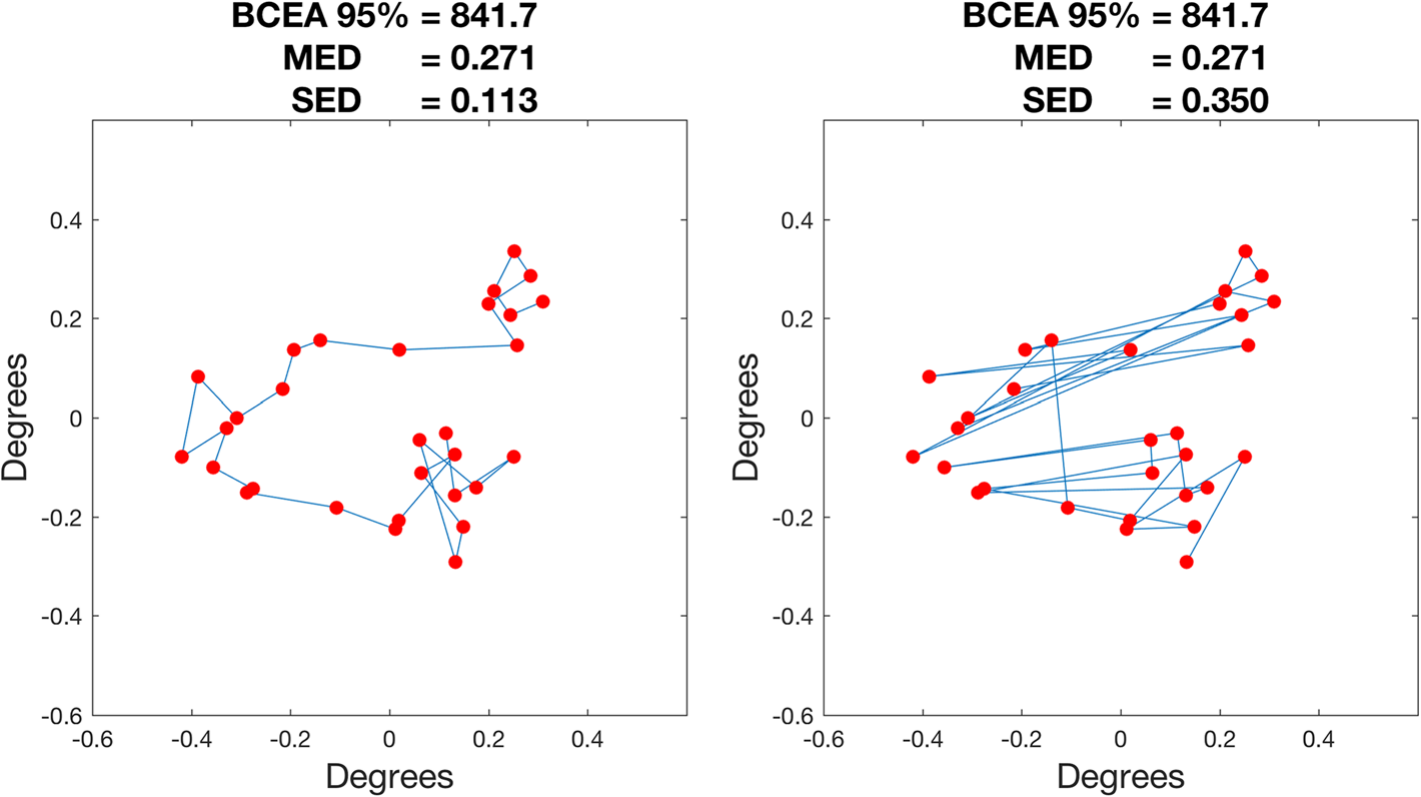
Schematic representation of the differences between the two proposed MED and SED indices. The left and right tracks represent two fictional fixation patterns (*n* = 30 points). The red dots represent the individual displacements of fixation and the blue lines join subsequent locations in the sequence. Both fixation patterns have the same point coordinates (the red dots) but the sequence is different. In the pattern on the left each displacement tends to be very close to the previous location in the sequence. In the pattern on the right, fixation shifts randomly from one point to another. As a result, indices that only account for fixation point locations (the BCEA and the MED) do not change. On the contrary, the SED index, calculated as the average of distances between successive points in the sequence, is greatly increased in the pattern on the right, capturing the continuous movement from one position to the other

In the present study, we aim to analyse fixation in patients with different severity of VF loss and people without VF loss (normal subjects) using data obtained during the Preferred Retinal Locus (PRL) registration performed prior to automated perimetry assessment in fundus perimetry. For this, we used a Compass perimeter (CenterVue, Padova, Italy), a recently introduced fundus perimeter for glaucoma testing [18, 19]. We aim to capture fixation information and relate it to measures of VF damage. We also propose two new measures of fixation instability that may better characterize the changes in fixation in glaucomatous patients (Fig. 1):

- The *Mean Euclidean Distance* (MED), which is a measure of central dispersion of fixation positions around the barycentre.
- The *Sequential Euclidean Distance* (SED), which is a measure of how frequently the subject is changing fixation location independently of the spatial spread of the points and is designed to encode the temporal instability of subjects’ fixation.

## Methods

### Data overview

The present study retrospectively analyses data from the validation study of the Compass perimeter [18]. The dataset contains 320 eyes of 320 people (200 normal subjects and 120 with a clinical diagnosis of glaucoma). All subjects underwent a standard ophthalmological evaluation and performed a visual field test using the Compass perimeter. This was carried out monocularly (random eye), using a 24–2 grid and a 4–2 staircase strategy.

### Inclusion/exclusion criteria

Details of inclusion and exclusion criteria for the study are reported in Rossetti et al. [18] Inclusion criteria for all subjects were: age between 20 and 80 years; best-corrected decimal visual acuity > 0.8 (for subjects < 50 years old) or > 0.6 (over 50) in both eyes; spherical refraction within ±5D; astigmatism within ±2D. For normal subjects: normal visual field in both eyes from Humphrey Field Analyzer (HFA) testing; normal appearance of the optic disc in both eyes; and an IOP ≤ 21 mmHg in both eyes. Glaucoma subjects were selected based on clinical diagnosis and structural damage to the optic nerve head (evident from fundus examination or OCT), independently of the visual field (i.e. preperimetric glaucoma subjects were included). Subjects with general or ocular conditions other than glaucoma that could affect visual field test results were not recruited. The study adhered to the tenets of the Declaration of Helsinki and all participants gave written informed consent. The local ethical committee (“San Paolo Hospital Ethics Committee”, n. 734 of July 30th, 2013—Studio GSD 2013) approved the original study and subsequent use of data in anonymized form for research purposes.

### PRL assessment and data extraction

Eye-tracking data were extracted from the Preferred Retinal Locus (PRL) assessment phase of the Compass perimeter. PRL assessment consists of a 10 s period prior to testing, during which time no stimuli are projected and the subject is asked to fixate the central target while retinal displacements are measured using an eye tracker. Eye tracking is performed using an infrared SLO picture of the subject’s retina with a temporal resolution of 25 times/sec. The theoretical spatial resolution is 0.03 degrees (derived from the 32 degrees/pixel resolution in the SLO picture) but may vary depending on the image quality. These data are used by the instrument to calculate the PRL for fixation, later set as the centre of the visual field testing grid.

The whole track of the PRL assessment consists of a list of horizontal and vertical displacements in degrees sampled every 40 ms (250 samples) along with a quality score of the tracking values provided by the instrument, based on the correlation coefficient between the fundus image at each time point and the reference image.

### Data analysis

All data were analysed in anonymized form. For each PRL assessment we selected only values with reliable tracking quality (Quality Score > 700). We excluded from our analysis subjects that had less than 150 reliable samples (< 60%). This resulted in 28 (8.75%) of the subjects being excluded (11 normal subjects and 17 glaucoma subjects).

For the remaining 292 participants, each participant’s data were processed independently to compute three metrics of fixation stability:

1. *Bivariate Contour Ellipse Area (BCEA):* BCEA is considered the current ‘Gold Standard’ measure of fixation stability [16] and is defined as an ellipse which encompasses fixation points for a given proportion of eye positions during one fixation trial. It assumes the distribution of the data to follow a Normal distribution along the two axes of the ellipse. Moreover, it only accounts for spatial alterations in the fixation pattern [17], ignoring the temporal sequence of the fixation displacements or simply the order of their occurrence.
2. The *Mean Euclidean Distance* (MED): MED is a measure of central dispersion of fixation positions around the mean position of the cloud of points (barycentre, see Fig. 1). It is computed by averaging the Euclidean distances of each tracked position from the location of the PRL. Differently from BCEA, it does not assume an elliptically shaped distribution of the points.
3. The *Sequential Euclidean Distance* (SED): SED is a measure of how frequently the subject is changing fixation location independently of the spatial spread of the points. It is computed by averaging the Euclidean distances of each tracked position from the subsequent location in the temporal sequence of displacements (see Fig. 1). Since some samples were excluded due to low tracking quality, gaps could appear in the temporal sequence. This issue was accounted for in the computation by measuring the distances only between temporally subsequent points both classified as reliable with the quality check.

The conceptual difference between the MED and SED is shown in the diagram in Fig. 1. BCEA 95% was calculated as reported in Crossland et al. [17].

As explained below (section on statistical analysis), log-transformed values of these indices were used for the analysis. They are denoted as log-BCEA, log-MED and log-SED respectively.

MATLAB (The MathWorks, Inc., Natick, Massachusetts, US) was used to extract the raw data and compute the various metrics.

### Visual (VF) field testing

**Fig. 2:**
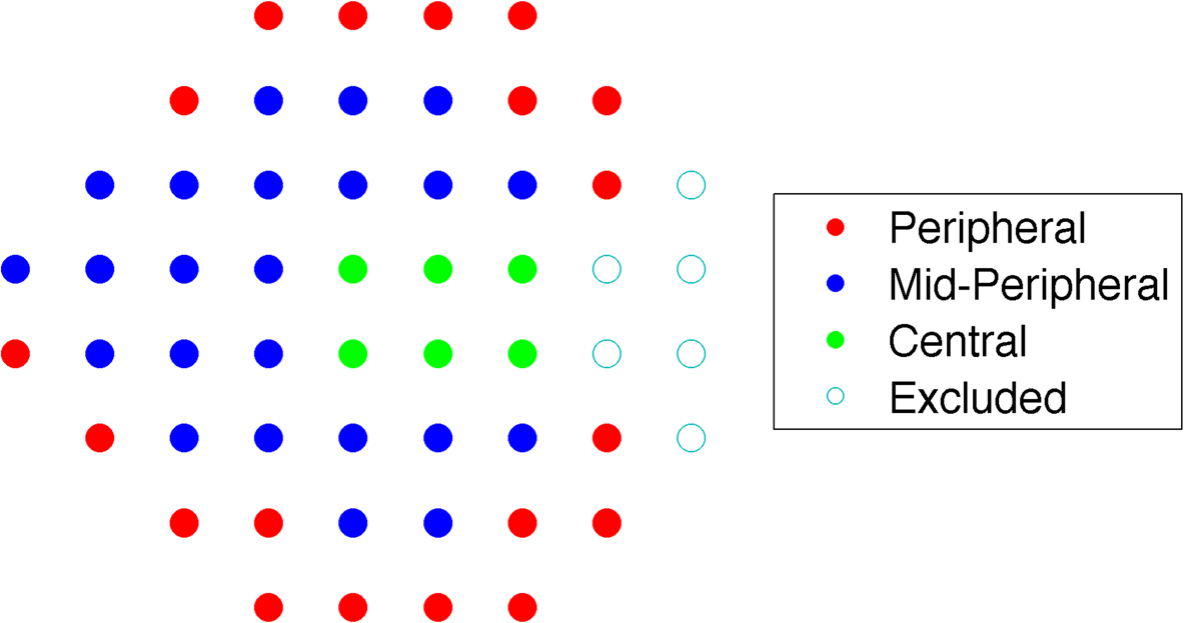
Garway-Heath Sectors. Schematic representation of the regional visual field subdivision used for the regional analysis. Sectors have been divided as proposed by Garway-Heath et al. and grouped into three regions, Peripheral, Mid-Peripheral and Central, according to their eccentricity, and regional MDs have been calculated by averaging the sector MD values in the same region. The temporal sector has been disregarded for this analysis

Mean Deviation (MD) and Pattern Standard Deviation (PSD) were calculated from visual field data. We also divided the visual field into sectors following a map described by Garway-Heath et al. [20] and calculated sector-wise MD values. Values from the temporal visual field sector were discarded and the remaining clusters were grouped in 3 categories (peripheral, mid-peripheral and central, see Fig. 2) to assess the local glaucoma visual field loss at increasing eccentricities.

### Statistical analysis

Although all calculations were performed in degrees (deg) for the MED and SED and in minutes of arc for the BCEA, we will report the indices as adimensional. The BCEA, MED and SED measures are strictly positive and exhibited a strong positive skew. To compensate for such skewness, their values were log-transformed prior to statistical analysis. The log-transformed measures are denoted as log-BCEA, log-MED andlog-SED respectively. The skewness of the residuals using log-transformed values was computed and evaluated. A skewness value outside the range − 0.5 and 0.5 was considered indicative of a non-symmetric distribution of the residuals [21].

We used linear models both to compare the control and glaucomatous groups and to analyse associations between fixation stability indices and the MD. All models included age as predictor to account for any possible changes in fixation with aging. To assess whether the age could have a non-linear effect on fixation metrics, we also fit the models using cubic basis splines, which are a common standard method to assess the presence of non-linear relationships [22]. Improvement in the goodness-of-fit with basis splines was evaluated using the Akaike Information Criterion (AIC) [22].

In order to assess which part of the visual field was affecting fixation the most when damaged, the correlation of fixation indices with the local MD values was analysed. In this analysis, we estimated a single model for each fixation index and used interactions to model different slopes for each visual field region (peripheral, mid-peripheral and central, see Fig. 2) and used the central region as the reference level. This was designed to investigate if damages to more peripheral parts of the visual fields would have an additional significant effect on the fixation indices. This could be considered to be a repeated measure design (with three regional MD values for each subject). Yet, the independent variable in this analysis was the same fixation index value (log-transformed BCEA, MED or SED) repeated three times, once for each regional MD value, yielding a perfect within subject correlation on the response variable. Therefore, since the variation of the response variable in each subject was equal to zero, this is the same as fitting a simple linear model, and no correction for repeated measures was used.

Model estimates are denoted as *ME*, Standard Errors as *SE* and Standard Deviations as *SD*.

All statistical analyses were carried out in R (https://www.r-project.org/).

## Results

Demographics for the study participants are reported in Table 1. Data from 292 subjects (189 normal subjects and 103 glaucoma subjects) were analysed (see Methods).

**Table 1:** Demographics of the final sample (*n* = 292). MD = Mean Deviation; PSD = Pattern Standard Deviation

	Normal	Glaucoma	*p*
Age	49.9 ± 15.21	71.14 ± 9.07	< 0.0001
MD	0.1 ± 1.38	−6.24 ± 6.93	< 0.0001
PSD	2.14 ± 0.56	6.1 ± 3.68	< 0.0001

Differences between the normal and glaucoma group are shown in Table 1. As expected we found a significant difference in the MD values (*P* <  0.001). Glaucoma patients were older on average (see Table 1) than the normal subjects; hence all analyses on the fixation indices included age as a covariate.

As shown in Table 2 (row 6), mean log-MED values appeared lower in glaucoma patients (− 1.47 ± 0.7) than controls (− 1.24 ± 0.86), and the non age corrected comparison yielded a statistically significant difference (*P =* 0.02). However, we did not observe a statistically significant difference in log-MED index once age was corrected for via multivariate analysis (estimated difference 0.16 ± 0.12, *ME ± SE*, *P =* 0.16, Table 2).

**Table 2:** Results of the group analysis of the fixation indices

	Normal	Glaucoma	*P*	Age corrected *P*
N° of Samples	229.8 ± 20.8	224.5 ± 24.73	0.053	0.18
BCEA	732.47 ± 778.97	1050.86 ± 950.22	0.002	
MED	0.31 ± 0.28	0.42 ± 0.4	0.005	
SED	0.07 ± 0.05	0.1 ± 0.06	< 0.0001	
log-BCEA	6.2 ± 0.88	6.52 ± 1.05	0.006	0.054
log-MED	− 1.47 ± 0.7	− 1.24 ± 0.86	0.02	0.16
log-SED	−2.84 ± 0.57	−2.48 ± 0.55	< 0.0001	0.002

*BCEA* Bivariate Contour Ellipse Area (at 95% in our study), *MED* Mean Euclidean Distance from the PRL, *SED* Sequential Euclidean Distance, *log-BCEA* log-transformed BCEA, *log-MED* log-transformed MED, *log-SED* log-transformed SED

We performed an equivalent analysis on the log-BCEA, with similar results. Log-BCEA values for the glaucoma group were higher and significantly different in the non age corrected comparison (*P =* 0.006), but we could not find a statistically significant effect in the age corrected multivariate analysis (estimated difference 0.28 ± 0.15, *ME ± SE*, *P =* 0.054, Table 2).

Then, sequential instability was measured with the log-SED index. Statistical analysis showed a significant difference in the non age corrected comparison (*P <* 0.0001), but, in contrast with the previous indices, a statistically significant effect could be identified even in the age corrected comparison (*P =* 0.002), glaucoma patients having a higher log-SED value (− 2.48 ± 0.55, Mean ± *SD*) compared to normal subjects (− 2.84 ± 0.57, Mean ± *SD*), yielding a 14.5% difference on the log-scale.

**Fig. 3:**
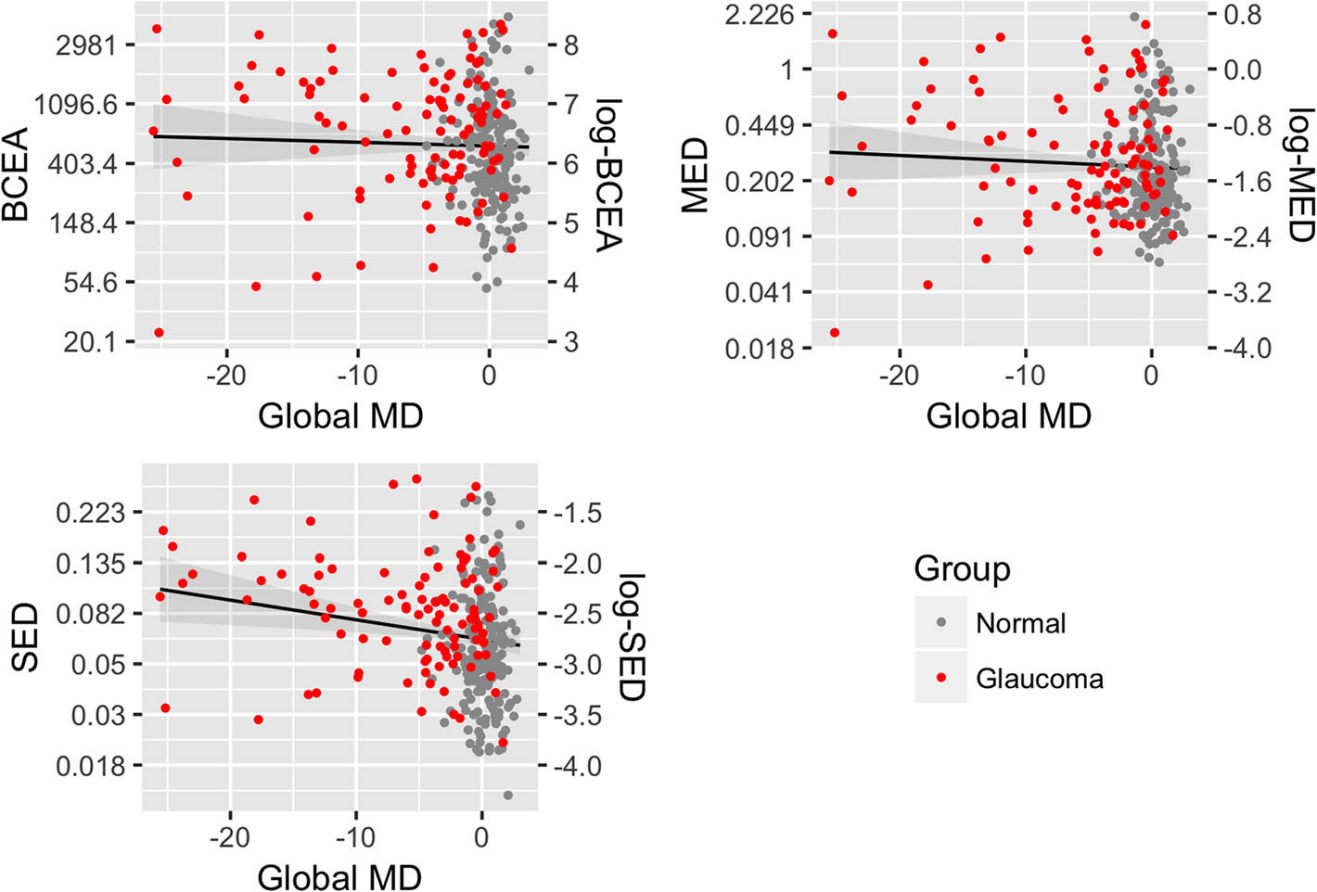
Fixation indices and MDs. Scatter plots and regression lines for the three fixation indices (log-BCEA, log-MED and log-SED) showing the relationship with the global MD value. Normal subjects are in green while glaucoma subjects are in red. The shaded region represents the 95% confidence interval of the estimate. The regression estimate was obtained from a multivariate model accounting for the age of the subject and the depicted line has been calculated at the mean age of the sample (57.4 years). The scale on the right vertical axis represents the values of the log transformed measures, while the left vertical axis reports the corresponding values in the linear scale

To analyse the relationship between fixation changes and the individual visual field loss, we also correlated the two indices with the MD, in a multivariate model with age as a covariate. The results are reported in Table 3. Both the log-BCEA and the log-MED were not significantly correlated with the MD values (all *P* > 0.14). In contrast the log-SED index had a highly significant association with the MD values (*P =* 0.00002). The results are depicted in the scatter plot in Fig. 3. We did not observe an improvement in the goodness-of-fit (i.e. reduction in the AIC) in any of the aforementioned models when evaluating non-linear effects of age on fixation metrics.

**Table 3:** Multivariate regression coefficients for global MDs

log-BCEA			log-MED			log-SED		
MD (SE)	Age (SE)	Intercept (SE)	MD (SE)	Age (SE)	Intercept (SE)	MD (SE)	Age (SE)	Intercept (SE)
− 0.006	0.006	6.295^***^	− 0.008	0.007^*^	6.268^***^	− 0.019^***^	0.005	6.227^***^
(0.012)	(0.004)	(0.061)	(0.009)	(0.004)	(0.395)	(0.007)	(0.004)	(0.093)

^*^p < 0.1; ^**^p < 0.05; ^***^p < 0.01

*SE* Standard Error, *MD* Mean Deviation, *log-BCEA* log-transformed BCEA, *log-MED* log-transformed MED, *log-SED* log-transformed SED

When analysing the correlation between the fixation indices and the local MD values for different regions of the visual field (peripheral, mid-peripheral and central, see Fig. 2), we found a significant correlation only for the log-SED and, as expected, the MD of the central region was the only significant predictor in the multivariate analysis (*P =* 0.005), with minimal non-significant contribution from the peripheral and mid-peripheral regions. The results are reported in Table 4. With marginal effect we refer to the effect that the change in the MD value of a non-central region has on the fixation indices when the MD of the central region is equal to 0. With additional effect we refer to the effect that the change in the MD value of a non central region has on the fixation indices when the MD of the central region is also affected. In this latter analysis, the goodness-of-fit was mildly increased for the log-SED and log-BCEA models when non linear effect of age was explored. However, this did not change the significance of the log-SED correlation and caused a very limited change in the estimate of its effect (− 0.016 ± 0.007, Mean ± SE, *p* = 0.03).

**Table 4:** Multivariate regression coefficients for regional MDs

	log-BCEA	log-MED	log-SED
Age (SE)	0.006^***^	0.005^***^	0.006^***^
	(0.002)	(0.002)	(0.001)
Central MD (SE)	− 0.002	− 0.003	− 0.020^***^
	(0.012)	(0.01)	(0.007)
Marginal effect of the mid-peripheral region (SE)	−0.016	−0.018	− 0.011
	(0.084)	(0.068)	(0.049)
Marginal effect of the peripheral region (SE)	−0.003	−0.006	− 0.005
	(0.084)	(0.068)	(0.049)
Additional effect of the mid-peripheral region (SE)	−0.006	−0.007	0.002
	(0.016)	(0.013)	(0.009)
Additional effect of the peripheral region (SE)	−0.001	− 0.002	0.004
	(0.016)	(0.013)	(0.009)
Intercept (SE)	6.305^***^	−1.392^***^	−2.748^***^
	(0.059)	(0.047)	(0.035)

^*^p < 0.1; ^**^p < 0.05; ^***^p < 0.01

*SE* Standard Error, *MD* Mean Deviation, *log-BCEA* log-transformed BCEA, *log-MED* log-transformed MED, *log-SED* log-transformed SED

The skewness of the residuals was within the critical range (− 0.5, 0.5) for all the aforementioned models.

## Discussion

This study retrospectively analysed eye-movement data obtained from the Compass perimeter in a large number of people with and without glaucoma. Fixation stability in glaucomatous subjects was shown to differ from normals when it was described by a measure of how frequently the subject is changing fixation location independently of the spatial spread of the points (log-SED). However, measures of stability based on traditional notions of dispersion (like BCEA and log-MED) did not show any significant differences. Furthermore, the log-SED better correlated with worsening visual field loss (measured with the MD) and better reflected the loss of the central visual field in the regional analysis. Taken as a whole, these findings indicate that glaucoma can affect some aspects of fixation, especially when the central visual field is involved, although not greatly altering the dispersion of fixation locations.

To our knowledge, only two previous papers have analysed fixation data measured with fundus perimetry in glaucoma patients, with mixed results [8, 9].

Longhin et al. analysed the differences between what they defined ‘static’ (during a pure fixation task) and ‘dynamic’ (during the microperimetric test) fixation, in four different diseases compared to normals, including POAG. In all groups they observed an increase in the BCEA values in the dynamic fixation compared to static fixation [9]. In our work we analysed data from the first 10 s of the examination, when no stimuli are projected and the subject is asked to fixate the central target for PRL detection. Therefore, our analysis is comparable to what they defined as ‘static’ fixation, i.e. during a pure fixation task. Longhin et al. reported stable fixation in 100% of the POAG subjects (based on the BCEA classification) in the ‘static’ fixation and did not perform formal testing to compare BCEA values in POAG to normal subjects. They did not report numerical values of the BCEA, but it can be deduced from the graphical depictions that BCEA values in POAG patients were not very different from those in normal subjects. This is in agreement with our results. Indeed we found only mild differences in the indices of pure fixation spreading (log-BCEA and log-MED) that were not statistically significant in the multivariate analysis corrected by age (see Table 2 and the Results section).

Shi et al. performed a more detailed analysis comparing glaucoma patients at different stages with normal subjects, analysing fixation data recorded during the microperimetric test; this procedure was analogous to the ‘dynamic’ fixation according to Longhin et al. They found a statistically significant effect when comparing the POAG patients with normal subjects in terms of number fixation points falling within the central 2 degrees (one degree around the foveal point) but not when comparing the number of points within the central 4 degrees [8]. They also reported a significant correlation with the sensitivity of the inferior temporal and superior temporal sectors [8]. These results might be explained by the fact that the central 2 degrees and the temporal sectors were the ones with the highest difference in sensitivity between normal subjects and glaucoma patients in their dataset [8]. In turn, this might simply be explained by glaucoma subjects in their specific sample not fixating with the more damaged parts of their visual fields. Moreover, since this analysis was performed on data acquired during the perimetric test, results might also be influenced by the gaze attraction towards more sensitive regions during the projection of the stimuli. In this perspective, although clearly showing that glaucoma can influence fixation, their results are more likely to reflect characteristics of the specific sample analysed, rather than general features of fixation in glaucoma patients.

In our analysis we found that, both in uncorrected and age corrected analysis, the log-SED index, which was designed to better capture the temporal fixation instability even in relatively restricted fixation areas, was significantly different in glaucoma patients compared to normal patients (see Table 2). This index of fixation instability was significantly correlated to the global MD (Table 3). When we analysed the correlation of the log-SED with the regional MD values, we found that the correlation with the global MD was mostly due to central impairment, with minimal non significant contributions from more peripheral regions of the visual field (Table 4).

These findings might be explained by patients with glaucoma typically not having a visual field loss starting in the foveal region and expanding peripherally. In contrast to macular degeneration, patients with glaucoma usually experience a progressive restriction of their central visual field, which is independent across the median raphe [23]. Therefore, an extremely widespread and displaced area of fixation points, as with retinal diseases, is not expected. This view is coherent with our data and data from the literature [8, 9] regarding the distribution of the fixation points, where POAG patients did not show strong differences from normal subjects when corrected by age.

Our working hypothesis is that patients with glaucoma might change the temporal features of their fixation, rather then exhibiting important fixation spread. This is reflected in the much stronger differences we have found when analysing the log-SED index. We speculate that these observations are coherent with the processing of visual information performed by the ganglion cells, which is more targeted to highlight variations rather than steady states [24]. Indeed, fixational eye movements have been involved in contrast sensitivity and stimulus detection, since they might prevent stimulus fading due to perceptual adaptation [24]. Moreover, in experimental setup on animal models, greater ganglion cell responses have been detected with wobbling stimuli compared to stationary stimuli [24]. Our results might reflect the idea of glaucoma patients trying to enhance the perception of the fixation target by frequent shifting between different positions. This idea would have to be tested further and this could be the subject of future work.

One limitation in the original database was that glaucomatous and normal subjects were not age matched. This feature was inherited from the study design and we accounted for that by using a multivariate analysis with age as a covariate. Such a mismatch might explain why we had different results when analyses were not age corrected. Non corrected analyses showed significantly increased values for all fixation metrics, including BCEA and log-MED, in glaucoma patients. This might be related to an age dependent effect on fixation features and highlights the importance of performing age corrected comparisons. From our analysis, the age effect on fixation indices appeared to be fairly linear, although some mild increase in the goodness-of-fit was noted when using basis spline in two of the models used to explore the effect of the local MD values. However, our analysis was not designed to test and analyse this aspect in particular, and modelling a non linear relationship with age only had minor effects on the estimates of the parameters of interest.

Future works could couple imaging data from the Compass perimeter with the structural information from OCT scans. In this way it might be possible to assess how local changes in the retinal nerve fibre layer and in the retinal ganglion cell complex might shape and modify the temporal and spatial pattern of fixation in glaucomatous patients, both during a pure fixation task and during the perimetric test. Indeed, Mallery et al. [25] recently showed that local ganglion cell loss is able to shape the fixation pattern in patients with optic neuropathies. Furthermore, other fixation stability parameters could be better derived from other statistical approaches such as Kernel Density Estimators [17, 25] and Brownian motion [26] modelling. In turn these might provide more information on the fine spatial and temporal modification of fixation in glaucoma damage.

## Conclusions

In conclusion, we provide evidence that in glaucoma subjects subtle changes in fixation are present, but are better described by features of the temporal sequence of displacements rather than by usual metrics of dispersion, like BCEA. Further investigations will be needed to understand the implications of this finding in visual tasks and perimetric testing.

## Abbreviations

BCEA: Bivariate Contour Ellipse Area
DLS: Differential light sensitivity
FAP: Fundus Automated Perimetry
HFA: Humphrey Field Analyzer
IOP: Intraocular pressure
MD: Mean Deviation
ME: Model estimates
MED: Mean Euclidean Distance
OCT: Optical Coherence Tomography
POAG: Primary Open Angle Glaucoma
PRL: Preferred Retinal Locus
SD: Standard Deviation
SE: Standard Error
SED: Sequential Euclidean Distance
SLO: Scanning Laser Ophthalmoscopy
VF: Visual field
